# Anatomy of the Facial Nerve at the Condylar Area: Measurement Study and Clinical Implications

**DOI:** 10.1155/2014/473568

**Published:** 2014-10-14

**Authors:** Hun-Mu Yang, Young-Bok Yoo

**Affiliations:** Department of Anatomy, College of Medicine, Dankook University, Cheonan, 119 Dandae-ro, Cheonan-si, Dongnam-gu, Chungnam 330-714, Republic of Korea

## Abstract

The aim of this study was to elucidate the detailed anatomy of the facial nerve (FN) at the condylar area to helping physicians preventing the iatrogenic trauma on the nerve. We dissected 25 specimens of the embalmed Korean cadavers (13 males and 2 females; mean age 76.9 years). The FN course at the condylar was examined, and the location of the FN branches was measured with superficial standards. The trunks of the FN emerged in the condylar area as one trunk, two trunks, and a loop or plexiform in 36%, 12%, and 52% areas, respectively. The zygomatic branch (Zbr) of FN passed over the tragus-alar line 23 mm anterior to the tragus (Tg) in most of the cases. The Zbr passed over the vertical line 2 cm anterior to the Tg through the area about 6 to 20 mm inferior to the Tg. Regardless of careful approach techniques to the condylar area, the FN could be damaged by a careless manipulation. Any reference landmarks could not guarantee the safety during the approach to the condylar area because more than half of the cases present the complicated branching type in the front of the Tg.

## 1. Introduction

The facial nerve (FN) emerges extracranially through stylomastoid foramen and then proceeds anteriorly as covered by the parenchymal tissues of the parotid gland. The FN is known to frequently bifurcate into two trunks at the retromandibular area and dividing five groups of twigs on the face: temporal (Tbr), zygomatic (Zbr), buccal (Bbr), marginal mandibular (Mbr), and cervical branches [1]. Since these branches innerve the facial expression muscles, traumas to the trunks or branches of the FN and viral infection resulting in swelling of the FN at the stylomastoid foramen can cause a facial palsy (Bell's palsy). Not only traumas or viral infection but also surgical interventions cause an iatrogenic paralysis of the facial expression muscles.

Surgical approaches have been performed in the preauricular area during the parotidectomy, reduction of the condylar fracture, decompression of the stylomastoid foramen for releasing the facial palsy, and the treatment of temporomandibular joint (TMJ) ankylosis [2, 3]. Pereira et al. reported that the preauricular incision for the surgical approaches would damage the FN at a risk up to 42.9% [4]. Anatomically, the Tbr, Zbr, and Bbr pass over the retromandibular area; the FN was vulnerable to be amputated during the preauricular incision. Thus, several studies have been focused on the prevention of the FN during surgical approaches. Narayanan et al. mentioned the nerve free window between the Bbr and Mbr at the distal portion of the nerve [3]. Because the branches diverged as proceeding distally, the anterior area on the parotid gland could function as enough nerve free area between the Bbr and Mbr to be approached safely. However, although the surgical approach can be attained along the nerve free window, the condylar portion of the retromandibular area would be manipulated during the reduction of the condylar fracture or the treatment of the TMJ ankylosis.

Laurentjoye et al. described that the Zbr and Bbr almost run along the tragus-alar line and the tragus-lip corner line, respectively [2]. However, several studies showed that the course of the FN was very complicated with communication among its branches. Yang et al. described that the FN consisted of not only distinct five thick trunks but also numerous branches on the face [5]. Kwak et al. reported that the FN bifurcated into two main branches in 86.7% area, and the branches of the bifurcation formed a loop after merging [1]. They also described that the Bbr was formed by a loop and anastomosed with Zbr and Mbr again. Considering them, its proceeding should be elucidated by the focused observation in the condylar area, and the metric information of its location should be provided with superficial landmarks.

The aim of this study is to elucidate the detailed anatomy of the FN at the preauricular area with superficial landmarks by meticulous dissections. We focused the proceedings of the FN condylar area and also followed other previous anatomical studies.

## 2. Methods and Materials

This study was performed in accordance with the Declaration of Helsinki. Twenty-five hemiface specimens were collected from 15 embalmed Korean cadavers (thirteen males and two females; mean age, 76.9 years). All the cadavers were legally donated to Dankook and Seoul National Universities. The facial skin at the parotid area concluding the condylar area was meticulously removed to reveal the subcutaneous tissue. The main trunks of the FN at the retromandibular area were exposed carefully without the displacement of the trunk by using alignment pin. After then, the ramified branches from the trunks were traced by removing the overlying subcutaneous tissues, the fascia, and parenchyma of the parotid gland.

**Figure 1:**
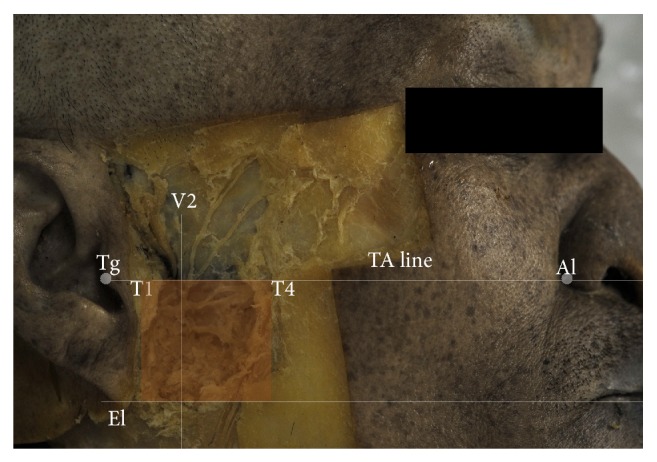
Reference lines and establishment of the condylar area. Tg: tragus; Al: ala of nose; El: lowermost point of earlobe; TA line: the line travelling between Tg and Al; V2: the line 2 cm anterior to the Tg and perpendicular to the TA line; T1: the point 1 cm anterior to the Tg on the TA line; T4: the point 4 cm anterior to the Tg on the TA line.

We demarcated the condylar area as the region vertically located between the tragus (Tg) and the lowermost point of the earlobe, horizontally located from 1 to 4 cm anterior to the Tg (Figure 1). The classification of the branching pattern of the FN was established. The cases presenting only one trunk of the FN issuing several branches was designated as Type I, and the cases presenting one trunk of the FN issuing the Tbr, Zbr, and Bbr and another trunk giving off the Bbr as proceeding parallel to the former were defined as Type II. Very complicated pattern showing a loop between the Zbr and Bbr or the plexiform nervous plexus in front of the Tg was established as Type III.

The measurements were taken to determine the location of the FN branches with reference to the superficial landmarks. We recruited two superficial reference lines for the measurements as follows:

1. TA line: the line proceeding between the Tg and alar points of the nose;
2. V2 line: the line perpendicular to the TA and located 2 cm anterior to the Tg.

The locations of the points where the FN branches met the TA line were examined by the observation on the verification of the TA line as a reference line for the Zbr at the condylar line. The locations of the points where the FN branches met the V2 were examined by the observation on the emerging position of the nerve. Digital calipers (catalogue number 500-196-20; Mitutoyo, Kanagawa, Japan) and the metric data were processed by means of Microsoft Excel 2007 (Microsoft Corp., Redmond, Wash.), and the metric data are presented as mean value ± standard deviation.

The condylar area was established as the red colored area which located vertically between the TA line and the line parallel to TA line through the El and horizontally between the vertical lines through T1 and T4.

## 3. Results

**Figure 2:**
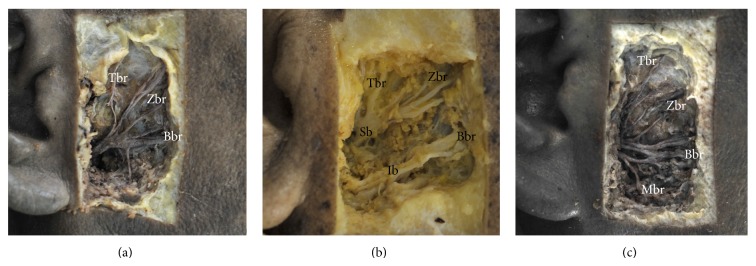
Three types of branching pattern of facial nerve at the condylar area. (a) Type I (single trunk), (b) Type II (double trunks), and (c) Type III (nervous loop or plexiform). Branches of facial nerve: Tbr: temporal branch; Zbr: zygomatic branch; Bbr: buccal branch; Mbr: marginal mandibular branch; Sb: superior trunk; Ib: inferior trunk.

The branching pattern of FN was classified into three types (Figure 2). In Type I (9 cases, 36%), single temporozygomaticobuccal trunk was found in the condylar area issuing the Tbr, Zbr, and Bbr (Figure 2(a)). The trunk giving off the Mbr and Cbr was not observed at the condylar area in this case. In Type II (3 cases, 12%), two distinct trunks were observed at the condylar area (Figure 2(b)). The upper branch gave off the Tbr, Zbr, and Bbr, and the lower trunk gave off several Bbrs proceeding parallel to the upper branch at intervals. The communicating branches between the upper and lower trunks were observed in most of these cases. In Type III (13 cases, 52%), the loop by Zbr and Bbr, or the nervous plexiform by the Zbr, Bbr, and Mbr was observed in the condylar area near the Tg (Figure 2(c)). All the branches of the FN emerged on the condylar area after passing through the area from the Tg and lowermost point of the earlobe.

The trunks of the FN passed over the V2 (the vertical line 2 cm anterior to the Tg) through one, two, and more than three points in 6 (24%), 12 (48%), and 7 cases (28%), respectively (Table 1). The trunk of the FN was proceeding through the point 17.6 ± 4.0 mm inferior to the Tg in the case of the trunk passing through only one point on the V2. In the case where the trunk passed through two points on the V2, the upper and lower trunks were proceeding through the points at 13.0 ± 4.3 and 20.0 ± 5.5 mm inferior to the Tg. In the case where the trunk passed through more than three points, the uppermost and lower point passing through the FN were located at 6.0 ± 2.8 and 20.9 ± 6.1 mm inferior to the Tg, respectively.

The several branches of the FN passed over the TA line at the condylar area, and the mean number of the branches was 2.1 (Table 2). The point where the Tbr passed over the TA line was located anterior to the Tg at 23 ± 5.7 mm. The minimal distance between the Tg and Tbr was 13.9 mm. The Zbr passed over the TA line in the condylar area in 18 cases (72%). The proceeding course of the Zbr at the condylar area in these cases was not parallel to the TA line. A single branch of Zbr passed over the TA line at the condylar area in eight cases (32%) through the point anterior at 36.7 ± 2.6 mm to the Tg. Double branches of the Zbr passed over the TA line at the condylar area in 10 cases (40%); upper branch of the Zbr passed over the TA line proximally through the point at 25.7 ± 4.1 mm anterior to the Tg, and the lower one passed over the line more distally through the point at 32.1 ± 4.2 mm.

## 4. Discussion

The present study revisited the FN pattern as focusing its distribution on the condylar area. With a convolution of the nervous distribution, the course of FN provided two discernible features with clinical consideration. (1) The loop and plexiform appearance of the nerve distribution in more than half necessitates to rethink a reckless direct approach to the condyle after preauricular incision. (2) Given that the Zbr mostly coursed over the TA line (tragus-ala line) at the condylar area, tragus-ala reference line would be useless near the mandibular condyle.

Although the manipulation of the condylar fracture is controversial among the maxillofacial surgeons, open reduction with internal rigid fixation cannot be excluded for the reasonable treatment providing the early mobilization and recovery of the joint function [6]. Several recommended incision techniques such as retromandibular and preauricular incision are reported. Tang et al. documented that the FN injury by the preauricular incision was reported at 3.2% to 42.9% and the injury by the submandibular incision at 5.3% to 48.1% [7]. Safe intervention for the mobilization can be manipulated by the anatomy of the condylar area in any incision technique. Surgical approach to the TMJ and rhytidectomy for the rejuvenation should be also performed with exact anatomical information about the FN in a vicinity of the mandibular condyle.

The condylar fractures occurred in about one third of the mandibular fractures [2]. This study established that the condylar area was located vertically between the Tg and the lowermost of the earlobe and horizontally between 1 and 4 cm from the Tg. This area was designated to include the mandibular condyle, condylar neck, and supra-angular area of the mandible considering that the condylar fracture and its reduction are supposed to be involved.

Kwak et al. reported that the main trunk of the FN bifurcated after proceeding anteroinferiorly was spread in 87% and the distance between the stylomastoid foramen and its furcation point was average of 13.0 mm [1]. After passing over the posterior border of the mandible, the FN ran anteriorly as two distinct trunks, temporozygomaticobuccal trunk and mandibulocervical trunk. The nerve free window was formed by the Bbr (the lowest branch of the temporozygomaticobuccal trunk) and the mandibulocervical trunk. In our study, all the branches in the condylar area passed over the area between the Tg and lowermost point of earlobe. The temporoparietal fascia and the parenchymal tissue of the parotid gland covered the FN branches. The pattern of this study did not indicate overall ramifying pattern but only showed the proceeding pattern at the condylar area. The common temporal zygomatic buccal trunk giving off the Tbr, Zbr, and Bbr was observed in Type I (36%) and two distinct trunks were observe in Type II (12%). In Type I, the mandibulocervical trunk proceeded below the lowermost point of the earlobe and the ramification point where the trunk issued Tbr, Zbr, and Bbr were located at the condylar area. Another trunk in Type II proceeded anteriorly forming the Bbr with or without the communication of tempozygomaticobuccal trunk. This Bbr from another trunk in Type II was susceptible to be damaged during the surgical approach to the condylar area along the nerve free window between the Bbr and Mbr. Moreover, the mean distance between the Zbr and Bbr at the point 3 cm anterior to the Tg was 9.4 mm, and the distance was <1 cm in 35.0% of all the types. Different from the nerve free window between the Bbr and Mbr, the area between the Zbr and Bbr in the condylar area was too narrow to be approached.

Laurentjoye et al. recommended the tragus-alar line tragus-lip corner line as landmarks of the Zbr and Bbr, respectively [2]. We also established the tragus-alar line as superficial reference line (TA). However, the simple reference line for the FA location is difficult to be established in the condylar area, whereas their suggestion would be reasonable at the distal part of the FA. In our study, the Zbr branches passed over the TA line in 18 cases (72%), and multiple branches of Zbr were proceeding over the TA line in 10 cases (40%). Moreover, the loop and plexiform of the FA branches were observed in the condylar area in Type III (52%), and thus the definite landmarks for the Zbr was hard to establish at the condylar area. Thus, the tragus-alar and tragus-lip corner lines do not guarantee the safety in the condylar area. The area ~23 mm anterior to the Tg on the TA line was regarded as the dangerous area.

One more distinct branch proceeded over the condylar neck in these areas. The branches of the FN at the condylar area passed over the V2 through the area from 6 to 20 mm inferior to the Tg. Ellis III reported that all the patients undergoing temporary atony after the FN injury recovered within six months [8]. Manisali et al. reported that six patients had temporary atony of the FN after the reduction of the condylar fracture, and none of them was permanent [9]. Regarding the proceeding of the FN in the condylar area, not only direct injury but also the pressure by swelling or retracted bone could cause temporary palsy. Narayanan et al. mentioned that slight injury on the anastomosis between the Zbr and Bbr could not cause the significant loss of function [3]. However, the thick trunk running over the condylar neck consisted of the motor component fiber to the various facial expression muscles [5]. Thus, significant injury on the FN in the condylar area, especially the branch over the condylar neck, can cause the facial expression muscles on broad area.

The incidence of the nervous anastomosis between the zygomatic and buccal branches is 87 to 100% [10, 11]. Laurentjoye et al. reported that the anastomoses between Zbr and Bbr were found between the temporal and cervicofacial branches in 65% [2]. Yang et al. reported very complicated anastomosis between the FA branches in all cases shown in the modified Sihler's staining [5]. Gosain proposed that Zbr and Bbr with higher number of nervous anastomosis were more robust than the Mbr with 15% anastomoses [12]. The number of twigs and nervous anastomosis in the condylar area is smaller than near or within the facial expression muscles. Therefore, the caution in the condylar area should be accompanied with the surgical approach regardless of the incision protocols because the FA injury on the proximal part at the condylar area would result in broad facial palsy.

## 5. Conclusion

The branches of the FN proceeding in the condylar area were located vertically between the Tg and lowermost point of the earlobe and horizontally between 1 and 4 cm anterior to the Tg. In more than half of the cases studied, the loop and plexiform of the FN branches were observed in the condylar area. The trunks passed over the condylar neck, ~0.5–2 cm inferior to the Tg. The tragus-alar line recommended as the reasonable reference line for the Zbr cannot guarantee the safety in the condylar area. Regardless of the incision technique, the FN on the condylar area could be damaged by reckless manipulation without anatomical information of the FN. This study could help to perform various safe surgical approaches useful in applied clinical anatomy.

## Figures and Tables

**Table 1:** Location of the facial nerve with 2 cm advanced to the tragus.

Number of intersecting points of the facial nerve and tragus-ala line	Distance between facial nerve and tragus-ala line (mm)
Meeting on one point (6 cases/24%)	17.6 ± 4.0
Meeting on two points (12 cases/48%)	
Upper point	13.0 ± 4.3
Lower point	20.0 ± 5.5
Meeting on three points (7 cases/28%)	
Uppermost point	6.0 ± 2.8
Lowermost point	20.9 ± 6.1

Data are mean ± SD values.

**Table 2:** Location of the facial nerve on the tragus-ala line.

Branch of facial nerve (average number of intersecting branches, 2.1)	Distance between facial nerve and tragus (mm)
Temporal branch (observed in all 25 cases)	23.8 ± 5.7
Zygomatic branch (in single intersection type, 8 cases/32%)	36.7 ± 2.6
Zygomatic branch (in double intersections type, 10 cases/40%)	
Anterior branch	25.7 ± 4.1
Posterior branch	32.1 ± 4.2

Data are mean ± SD values.
